# LDLR Gene Polymorphisms (rs5925 and rs1529729) Are Associated with Susceptibility to Coronary Artery Disease in a South Indian Population

**DOI:** 10.3390/medsci7070080

**Published:** 2019-07-15

**Authors:** Chandan K. Jha, Rashid Mir, Imadeldin Elfaki, Shaheena Banu, S. M. S. Chahal

**Affiliations:** 1 Department of Human Genetics, Punjabi University, Punjab 147002, India; 2 Department of Medical Lab Technology, Faculty of Applied Medical Sciences, University of Tabuk, Tabuk 71491, Saudi Arabia; 3 Department of Biochemistry, Faculty of Science, University of Tabuk, Tabuk 71491, Saudi Arabia; 4 Sri Jayadeva Institute of Cardiovascular Science and Research, Bangalore 560069, India

**Keywords:** cardiovascular diseases (CVD), allele-specific-PCR, lipoprotein receptor (LDLR), atherosclerosis, SNPs rs5925 and rs1529729, hypercholesterolemia

## Abstract

Cardiovascular diseases (CVD) are a major cause of death in India and worldwide. Atherosclerosis is caused by the interaction of environmental and genetic factors. Hypercholesterolemia is an example of a classical risk factor for CVD. The low-density lipoprotein receptor (LDLR) is one of the regulating mechanisms the liver uses for cholesterol homeostasis. Gene variations in the LDLR have been reported to cause hypercholesterolemia and consequently CVD. We investigated the association of polymorphisms in the LDLR (rs5925 and rs1529729) with coronary artery disease (CAD) in 200 coronary artery disease patients and 200 matched healthy controls using allele-specific PCR (AS-PCR). The results indicated that the CT genotype of the rs1529729 polymorphism was associated a decreased susceptibility to CAD with an odds ratio (OR) = 0.42 (95% confidence interval (CI), 0.23–0.77), risk ratio (RR) = 0.59 (0.39–0.89), *P* = 0.0047. The TT genotype of the rs1529729 polymorphism was also associated with decreased susceptibility to CAD with an OR = 0.19 (95% CI, 0.076–0.47), RR = 0.57 (0.47–0.69), *P* = 0.0003. The GA genotype of the rs5925 polymorphism was associated with decreased susceptibility to CAD with an OR = 0.45 (95% CI, 0.27–0.75), RR = 0.65 (0.47–0.88), *P* = 0.002. We concluded that the CT and TT genotypes of the rs1529729 polymorphism and the GA genotype of the rs5925 polymorphism are probably associated with decreased susceptibility to CAD. The simplicity of AS-PCR makes it particularly suitable for the rapid, large-scale screening of gene variabilities in the LDLR. AS-PCR could provide significant benefits in clinical applications with its ability to amplify a lower quantity of samples in a cost-saving manner. Nevertheless, these findings need to be validated in well-designed studies with larger sample sizes and in different populations.

## 1. Introduction

Coronary artery disease (CAD) is a complex disease resulting from the interaction of genetic and environmental factors. Traditional risk factors for atherosclerosis include obesity, hypercholesterolemia, smoking, hypertension, and hyperglycemia [1]. These factors lead to an excessive accumulation of cholesterol, which results in hardening of and accumulation of thrombotic debris in the artery wall [2]. The steps involved in the formation of an atherosclerotic lesion begin with an injury to the endothelial wall, after which the retention of lipid particles occurs, followed by inflammation. These steps lead to the generation of a necrotic core (containing cell debris and lipids) covered by a fibrous cap, eventually leading to the formation of an atheromatous plaque [3]. Hypercholesterolemia is one of the important risk factors involved in the formation of atherosclerotic plaques [4]. It has been reported that the deposition of cholesterol particles in the endothelial wall initiates the inflammatory response, which involves the activation of macrophages and lymphocytes, as well as the production of cytokines (including tumor necrosis factor-alpha, interleukin-6, and interferon-gamma) [5], and enhances the development of atheroma [4]. Cholesterol is pooled in the liver from the diet or from cholesterol that is synthesized by cells. The liver is the primary organ for the regulation of cholesterol homeostasis, and the low-density lipoprotein receptor (LDLR) is one of the regulating mechanisms [6]. The LDLR is a transmembrane glycoprotein that plays an important role in the uptake of low-density lipoprotein (LDL) from the blood circulation in a process that is mediated by apolipoprotein B [7,8]. The LDLR binds at neutral pH specifically and with a high affinity to extracellular lipoprotein particles [9]. The LDLR and LDL–cholesterol complex is then brought into the cell by endocytosis [10]. LDL–cholesterol is then released by the LDLR at an acidic pH for degradation by a lysosome which results in the release of free cholesterol and the return of the LDLR to the cell surface [9]. Genome-wide association studies (GWASs) have discovered certain novel gene loci that reproducibly associate with diseases [11,12,13], including CAD [14,15,16,17,18,19,20] and atherosclerosis [21,22]. Mutations in the *LDLR* gene have been reported to cause familial hypercholesterolemia [18,23]. In the present study, we investigated the association of polymorphisms in the LDLR (rs5925 and rs1529729) and CAD in a cohort from the Bangalore population.

## 2. Subjects and Methods

This project has been approved by the institutional ethics committee (IEC), Punjabi University, Patiala, project No. 268/DLS/HG. We conducted a population-based case–control study including 200 patients with clinically confirmed CAD and 200 healthy controls (HC) with no history of CAD and no familial relationship to the CAD patients. We excluded any patient with a previous history of chronic disease from this study.

### 2.1. Collection of Blood Samples and Clinical History

About 3 mL of peripheral blood was collected in an EDTA-containing vial from each patient and healthy control after they completed a questionnaire. We collected information as well as an informed written consent form from both CAD patients and healthy controls regarding personal information such as name, gender, and age. Additionally, information regarding a history of sexually transmitted diseases and addiction, such as smoking and alcohol, were collected. We also collected laboratory and clinical data.

### 2.2. Extraction of DNA

DNA was extracted from the blood using the modified glass bead method, as described in a previous study [24]. The extracted DNA was dissolved in 100 μL of 10 mM Tris-Cl (pH 8.0) buffer and stored at 4 °C until use. The quality of the DNA was assessed by gel electrophoresis.

### 2.3. Genotyping of the LDLR Polymorphisms (rs5925 and rs1529729)

**Figure 1:**
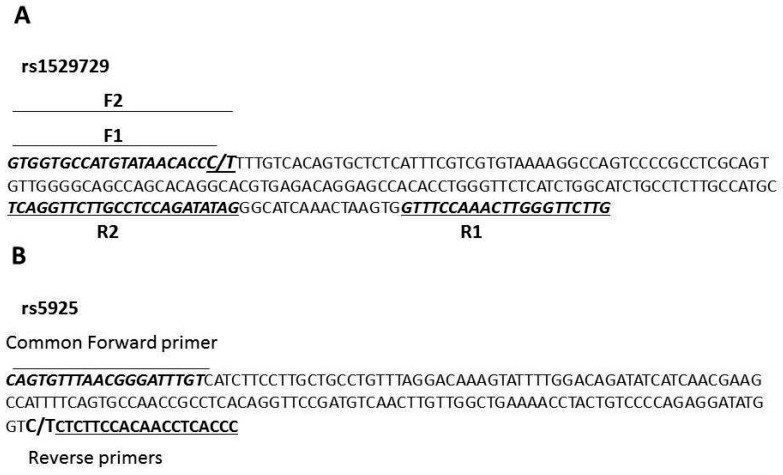
The annealing sites of the primers used for genotyping rs1529729 (**A**) and rs5925 (**B**).

**Figure 2:**
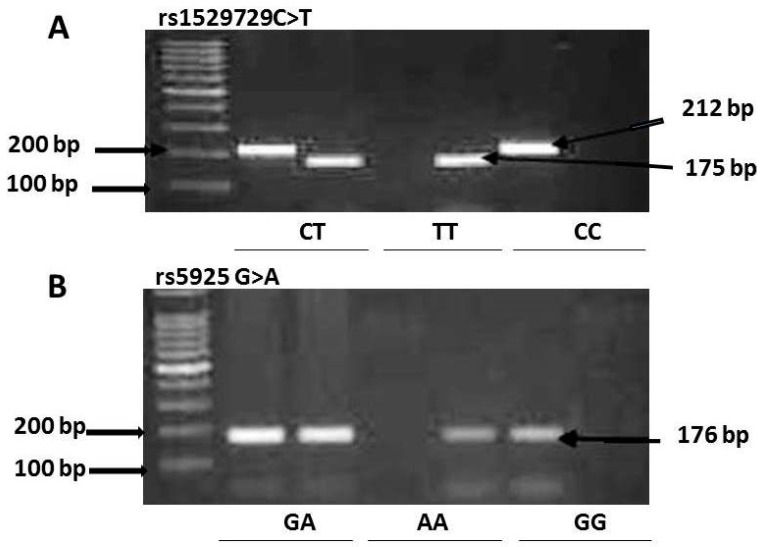
Genotyping of rs1529729 C > T (**A**) and rs5925 G > A (**B**) polymorphisms using allele-specific PCR (AS-PCR) assay.

Gene polymorphisms were detected using allele-specific PCR (AS-PCR). AS-PCR is based on the use of sequence-specific PCR primers that allow for amplification of the template DNA when the target allele is contained within the sample. Primers were designed using primer 3 software (Table 1, Figure 1). For rs5925, AS-PCR was performed in two tubes with each of the tubes containing a common forward primer and a different reverse primer. The reaction mixtures for the rs5925 AS-PCR contained template DNA, 3–4 μL (50 ng); the common forward primer, 0.3 μL (25 pmol); a reverse primer, 0.3 μL (25 pmol); Coral load dye, 2.5 μL; 12.5 μL of TopTaq Master Mix (Qiagen, Germany); and enough nuclease free ddH_2_O to bring the final volume to 25 μL. The AS-PCR for rs1529729 was performed in two tubes, each containing a different primer set. The reaction mixture for the rs152972 AS-PCR contained DNA template, 3–4 μL (50 ng); either the F1/R2 or the F2/R1 primer combinations, 0.3 μL of each primer (25 pmol); Coral load dye, 2.5μL; 12.5 μL of TopTaq Master Mix (Qiagen, Germany); and enough nuclease free ddH_2_O to bring the final volume to 25 μL. The PCR conditions used were as follows: initial denaturation for 10 min at 95 °C, 35 cycles of 30 s at 95 °C (denaturation), 30 s at 57 °C (the rs5925 AS-PCR) or 61 °C (the rs1529729 AS-PCR) (annealing), and 1 min at 72 °C (elongation), followed by 10 min at 72 °C (final elongation). The PCR products were visualized using electrophoresis via 2% agarose gel stained with ethidium bromide (Figure 2). The lengths of the PCR products for rs1529729 were 212 bp for F1/R1, and 175 bp for F2/R2 PCR products, and 176 bp for the rs5925 (Figure 2).

### 2.4. Statistical Analysis

Group differences were compared using a Student’s two-sample *t*-test or a one-way analysis of variance (ANOVA) for continuous variables, and a Chi-square test for categorical variables. Differences in both the single nucleotide polymorphism SNP allele and in the genotype frequencies between groups were evaluated using the Chi-square test. The associations between both SNP genotypes and the risk of CAD were estimated by computing the odds ratios (ORs), risk ratios (RRs), and risk differences (RDs) with 95% confidence intervals (CIs). Allele frequencies among cases, as well as controls, were evaluated using the Chi-square test. *P* < 0.05 was considered significant. All statistical analyses were performed using SPSS 16.0 (IBM, Chicago, IL, USA).

## 3. Results

A total of 200 CAD patients and 200 healthy controls were included in this study. The demographic characteristics of CAD patients and controls are shown in Table 2. The ratios of gender and age differences in CAD patients are comparable to those of the control group. The clinical characteristics of the CAD patients are shown in Table 3.

### 3.1. The Genotype Frequency of the LDLR Polymorphisms rs1529729 and rs5925

The genotype frequency of the rs1529729 polymorphisms CC, CT, TT in patients were 9, 77, and 14%, respectively, whereas they were 21, 76, and 3% in controls, respectively. The differences in the proportions of the genotype frequencies were significantly different (*P* = 0.0001, Table 4). The genotype frequency of the rs5925 polymorphisms GG, GA, AA in patients were 27, 62, and 11% respectively, whereas they were 15, 76, and 9% in controls, respectively. The differences in the proportions of the genotype frequencies were significantly different (*P* = 0.006, Table 4).

### 3.2. rs1529729 C > T and rs5925 G > A Polymorphisms Were Associated with CAD

The results of the present study indicated that in the codominant model the CT genotype of the rs1529729 polymorphism was associated with a decreased susceptibility to CAD with an OR = 0.42 (95% CI, 0.23–0.77), RR = 0.59 (0.39–0.89), *P* = 0.0047. The TT genotype was also associated with a reduced risk for CAD with an OR = 0.09 (95% CI, 0.03–0.26), RR = 0.36 (0.24–0.55), *P* = 0.0001 (Table 5). In the dominant model the CT + TT genotype was associated with a decreased susceptibility to CAD with an OR = 0.37 (95% CI, 0.21–0.67), RR = 0.56 (0.38–0.84), *P* = 0.001. The TT genotype was associated with decreased susceptibility to CAD with OR = 0.19 (95% CI, 0.076–0.47), RR = 0.57 (0.47–0.69), *P* = 0.0003 (Table 5).

Our results also showed that in the codominant model the GA genotype of the rs5925 polymorphism was associated with a decreased susceptibility to CAD, OR = 0.45 (95% CI, 0.27–0.75), RR = 0.65 (0.47–0.88), *P* = 0.002. In the dominant model the GA + AA genotype was associated with a reduced risk of CAD with OR = 0.477 (95% CI, 0.28–0.78), RR = 0.66 (0.48–0.9), *P* = 0.003 (Table 5). Our results also showed that covariates such as gender, age, blood levels of random sugar, total cholesterol, low-density lipoprotein cholesterol (LDL-C), and high-density lipoprotein cholesterol (HDL-C) were non-significantly different (*P* > 0.05) among the genotypes of both the SNPs in the patient group. We also did not see significant effects (*P* > 0.05) of diabetes, hypertension, intake of alcohol, smoking, and pan masala on either the rs5925 or the rs1529729 polymorphisms (Table 6). These results were unexpected and might be due to the limited sample size used in this research.

## 4. Discussion

### 4.1. Association of rs1529729 C > T and rs5925 G > A Genotypes with CAD

Cardiovascular disease (CVD) represents an economic and health burden all over the world [25]. CVD has become a leading cause of death in all parts of India. In India, CVD has increased by 59%, from 23.2 million (1990) to 37 million (2010) [26]. One thousand and seven hundred mutations in the *LDLR* gene have been associated with familial hypercholesterolemia [23], which is one of the traditional risk factors for CVD [27]. This fact has prompted us to examine the association of the LDLR rs1529729 C > T and rs5925 G > A gene variations with CAD. Our results indicated that the rs1529729 C > T genotype distribution is different between the cases and the control (*P*-value = 0.0001, Table 4). Moreover, our results showed that the CT and TT genotypes of rs1529729 C > T are associated with decreased susceptibility to CAD with an OR = 0.42 (95% CI, 0.23–0.77), RR = 0.59 (0.39–0.89), *P* = 0.0047, and an OR = 0.09 (95% CI, 0.03–0.26), RR = 0.36 (0.24–0.55), *P* = 0.0001, respectively (Table 5). At the allelic level, the T allele is associated with a reduced susceptibility to CAD with an OR = 0.63 (95% CI, 0.47–0.83), RR = 0.79 (0.69–0.91), *P* = 0.0011 (Table 5). We did not see significant differences in the random blood sugar (RBS), triglyceride, cholesterol, HDL-C, and LDL-C levels between the rs1529729 genotypes in CAD patients (*P*-value > 0.05, Table 6). This may be due to the relatively small sample size taken in this study. These results may be in good agreement with the study by Kathiresan et al., 2008 [28].

The results showed that the rs5925 G > A genotype distribution is different between the cases and the control (*P*-value = 0.006, Table 4). It was indicated that the GA genotype of the rs5925 polymorphism is associated with decreased susceptibility to CAD with an OR = 0.45 (95% CI, 0.27–0.75), RR = 0.65 (0.47–0.88), *P* = 0.002. The rs5925 polymorphism (in cooperation with the rs688 polymorphism) has been shown to regulate the splicing efficiency of the *LDLR* gene [29]. This result may be consistent with a study that showed that the rs5925 polymorphism is associated with the thickness of the carotid-intima media in Slovenian type 2 diabetes T2D patients [30]. Furthermore, the rs5925 polymorphism has been predicted to be one of the SNPs that cause familial hypercholesterolemia in the Malaysian population [31].

Our results also showed that there are no significant differences (*P* > 0.05) between the rs5925 genotype distribution and RBS, triglycerides, cholesterol, HDL-C, and LDL-C levels (Table 6). Again, these results may be due to the small sample size, or perhaps some of the CAD patients had been treated with hypolipidemic agents. LDLR is a transmembrane glycoprotein at the hepatocyte surface that plays an important role in cholesterol homeostasis [8]. We suggest that the T allele of the rs1529729 polymorphism and the GA genotype of the rs5925 polymorphism protect against CAD by increasing the expression of LDLR at the hepatocyte surface such that LDL-C uptake and metabolism is enhanced. In support of this suggestion, the rs5925 polymorphism has been described as an exon-splicing enhancer [29]. However, the effect of rs1529729 and rs5925 polymorphisms on LDLR expression should be to be elucidated in a future study. 

To our knowledge, this is the first study that has shown the potential associations of the rs5925 and rs1529729 polymorphisms with CAD in a South Indian population. The limitations of this study include a relatively small sample size and the fact that the study population contained a high percentage of males compared to females (Table 2).

### 4.2. The Frequency of the rs5925 and rs1529729 Polymorphisms in Different Populations

The frequency of the rs5925 genotypes GG, GA, and AA has been studied in different populations (Table 7). The frequency of the rs1529729 genotypes CC, CT, and TT have been reported in an Iranian population as 28.43, 42.16, and 29.41%, respectively (Table 7). In the present study, the rs1529729 genotype distributions were 21, 76, and 3% (Table 7). This difference may be due to the different sample size or different ethnicity.

The results showed that the lowest percentage of the GG genotype in controls was (4%) in the Taiwanese population, while the highest was (56.5%) in the Chinese population (Table 7). Our study found that the GG genotype in controls was 15%, which is consistent with previous findings (Table 7). The GA genotype ranged from 51 to 34% in Mexican and Taiwanese populations, respectively. The GA genotype in our study was relatively high (76%). In this study, the AA genotype in the control group was 9%, which is within the range of previous findings (8 to 62%) in Chinese and Taiwanese populations, respectively (Table 7).

## 5. Conclusions

Taken together, the results of the present study indicated that the CT and TT genotypes of the rs1529729 polymorphism and the GA genotype of the rs5925 polymorphism are associated with decreased susceptibility to CAD in a South Indian population. However, these results must await further validation in future studies with larger sample sizes and in different populations. Moreover, a proteomic study on the effect of the rs1529729 and rs5925 polymorphisms on the LDLR protein is recommended.

## Figures and Tables

**Table 1:** Primers sequences of allele-specific (AS)-PCR used for genotyping the low-density lipoprotein receptor (LDLR) gene polymorphisms rs1529729 and rs5925.

SNP		Primer Sequence	Product Size	T_a_
**rs1529729**	F1 Forward primer (C allele)	5-GTGGTGCCATGTATAACACCC-3	175 bp	61 °C
	R1 Reverse primer	5-CAAGAACCCAAGTTTGGAAAC-3		
	F2 Forward primer (T allele)	5-GTGGTGCCATGTATAACACCT-3	212 bp	
	R2 Reverse primer	5-CTATATCTGGAGGCAAGAACCTGA-3		
**rs5925**	Reverse primer (G allele)	5′-GGGTGAGGTTGTGGAAGACG-3′	176 bp	57 °C
	Reverse primer (A allele)	5′-GGGTGAGGTTGTGGAAGACA-3′	176 bp	
	Common Forward primer	5′-CAGTGTTTAACGGGATTTGT-3′		

**Table 2:** Demographic characteristics of coronary artery disease (CAD) patients and healthy controls.

Variables	No. of CAD Cases (*n* = 200 (100%)	No. of Healthy Controls (*n* = 200 (100%)
No. of cases and controls	200 (100%)	200 (100%)
**Gender difference**		
Males	180 (90%)	176 (88%)
Females	20 (10%)	24 (12%)
**Age difference**		
Age ≤50	90 (45%)	88 (44%)
Age >50	110 (55%)	112 (56%)

**Table 3:** Baseline characteristics of CAD patients.

Variables	CAD Cases (*n* = 200)	(%)
**Random blood sugar (RBS)**		
≤140 mg	129	(64.5%)
>140 mg	71	(35.5%)
**Cholesterol**		
≤200 mg	176	(88%)
>200 mg	24	(12%)
**High-density lipoprotein cholesterol (HDL-C)**		
≤40 mg	166	(83%)
>40 mg	34	(17%)
**Low-density lipoprotein cholesterol (LDL-C)**		
≤100 mg	150	(75%)
>100 mg	50	(25%)
**Triglycerides (TGL)**		
≤150 mg	105	(52.5%)
>150 mg	95	(47.5%)
**Coronary heart disease (CHD) in family**		
Yes	15	(7.5%)
No	185	(92.5%)
**Hypertension**		
Yes	29	(14.5%)
No	171	(85.5%)
**Type 2 diabetes**		
Yes	39	(19.5%)
No	161	(80.5%)
**Smoking**		
Yes	121	(60.5%)
No	79	(39.5%)
**Alcohol**		
Yes	71	(35.5%)
No	129	(64.5%)
**Pan masala**		
Yes	4	(2%)
No	196	(98%)

**Table 4:** The genotype frequency of the *LDLR* polymorphisms of study cohorts (controls and CAD patients).

SNP	Genotype	C/C	C/T	T/T	Chi-Square	Df	*P*-Value
rs1529729	CAD patients *n* = 200 (%)	18 (9%)	154 (77%)	28 (14%)	23.85	2	0.0001
	Controls *n* = 200 (%)	42 (21%)	152 (76%)	06 (3%)			
rs5925	Genotype	G/G	G/A	A/A	Chi-square	Df	*P*-value
	CAD patients *n* = 200 (%)	54 (27%)	124 (62%)	22 (11%)	10.1	2	0.006
	Controls *n* = 200 (%)	30 (15%)	152 (76%)	18 (9%)			

**Table 5:** Association of the *LDLR* rs1529729 C > T and rs5925 G > A gene variations with CAD.

SNP	Genotypes	Healthy Controls	CAD Cases	Odds Ratio (OR) (95% CI)	Risk Ratio (RR)	*P*-Value
**rs1529729**		(*n* = 200)	(*n* = 200)			
	**Codominant**					
	LDLR-CC	42	18	**1 (ref.)**	**1 (ref.)**	
	LDLR-CT	152	154	0.42 (0.23–0.77)	0.59 (0.39–0.89)	0.0047
	LDLR-TT	06	28	0.09 (0.03–0.26)	0.36 (0.24–0.55)	0.0001
	**Dominant**					
	LDLR-CC	42	18	1 (ref.)	1 (ref.)	
	LDLR-(CT + TT)	158	182	0.37 (0.21–0.67)	0.56 (0.38–0.84)	0.001
	**Recessive**					
	LDLR-(CC + CT)	194	172	1 (ref.)	1 (ref.)	
	LDLR-TT	06	28	0.19 (0.076–0.47)	0.57 (0.47–0.69)	0.0003
	**Allele**					
	LDLR-C	236	190	1 (ref.)	1 (ref.)	
	LDLR-T	164	210	0.63 (0.47–0.83)	0.79 (0.69–0.91)	0.0011
**rs5925**	**Codominant**					
	LDLR-GG	30	54	1 (ref.)	1 (ref.)	
	LDLR-GA	152	124	0.45 (0.27–0.75)	0.65 (0.47–0.88)	0.002
	LDLR-AA	18	22	0.67 (0.32–1.46)	0.79 (0.50–1.24)	0.322
	**Dominant**					
	LDLR-GG	30	54	1 (ref.)	1 (ref.)	
	LDLR-(GA+AA)	170	146	0.477 (0.28–0.78)	0.66 (0.48–0.9)	0.003
	**Recessive**					
	LDLR-(GG+GA)	182	178	1 (ref.)	1 (ref.)	
	LDLR-AA	18	22	1.24 (0.64–2.4)	1.12 (0.78–1.6)	0.5
	**Allele**					
	LDLR-G	212	232	1 (ref.)	1 (ref.)	
	LDLR-A	188	168	0.8 (0.61–1.07)	0.9 (0.78–1.03)	0.107

**Table 6:** Correlations of the covariates with rs5925 and rs1529729 genotypes.

rs1529729								rs5925					
Subjects	*n* = 200	C/C	C/T	T/T	X^2^	DF	*P* value	G/G	G/A	A/A	X^2^	DF	*P*-value
**Correlation with gender**													
Males	180	15	138	27	2.2	2	0.33	48	111	21	0.83	2	0.66
Females	20	03	16	01				06	13	01			
**Correlation with age**													
Age ≤ 50	90	07	73	10	1.61	2	0.447	23	58	09	0.43	2	0.806
Age > 50	110	11	81	18				31	66	13			
**Correlation with RBS**													
RBS ≤ 140 mg	129	13	98	18	0.52	2	0.77	33	83	13	0.87	2	0.647
RBS > 140 mg	71	05	56	10				21	41	09			
**Correlation with cholesterol**													
Cholesterol ≤ 200 mg	176	14	137	25	1.96	2	0.375	45	114	17	5.33	2	0.069
Cholesterol > 200 mg	24	04	17	03				09	10	05			
**Correlation with HDL**													
HDL ≤ 40 mg	166	14	128	24	0.5	2	0.778	44	104	18	0.18	2	0.913
HDL > 40 mg	34	04	26	04				10	20	04			
**Correlation with LDL**													
LDL ≤ 100 mg	150	15	113	22	1.07	2	0.5857	44	92	14	2.77	2	0.25
LDL > 100 mg	50	03	41	06				10	32	08			
**Correlation with TGL**													
TGL ≤ 150 mg	105	10	79	16	0.4	2	0.8187	25	66	14	1.95	2	0.377
TGL > 150 mg	95	08	75	12				29	58	08			
**Correlation with hypertension**													
Hypertension	29	03	22	04	0.07	2	0.9656	04	22	03	3.26	2	0.195
No hypertension	171	15	132	24				50	102	19			
**Correlation with diabetes**													
Diabetes	39	04	30	05	0.13	2	0.9371	07	28	04	2.24	2	0.326
No diabetes	161	14	124	23				47	96	18			
**Correlation with CHD**													
CHD	15	02	11	02	0.37	2	0.8311	01	12	02	3.41	2	0.181
No CHD	185	16	143	26				53	112	20			
**Correlation with smoking**													
Smoking	121	13	93	15	1.6	2	0.4493	26	45	08	2.32	2	0.313
No smoking	79	05	61	13				28	79	14			
**Correlation with alcohol**													
Alcohol	71	8	52	11	1.01	2	0.6035	18	47	06	1.07	2	0.585
No alcohol	129	10	102	17				36	77	16			
**Correlation with pan masala**													
Pan masala	04	00	04	00	1.22	2	0.5434	00	03	01	1.94	2	0.379
No pan masala	196	18	150	28				54	121	21			

**Table 7:** The rs5925 G > A and rs1529729 C > T genotype distributions in different populations.

rs5925									
**Country**	**Disease**	***n***	**Homozygous Wild Type**	**%**	**Heterozygous**	**%**	**Homozygous Mutant**	**%**	**Reference**
Mexico	Hypertension	160	36	22.5	73	45.63	51	31.87	[32]
	Controls	160	34	21.25	82	51.25	44	27.5	
Slovenia	Type 2 diabetes	399	67	16.8	189	47.4	143	35.8	[30]
	Controls	196	26	13.3	91	46.4	79	40.3	
Taiwan	Ischemic stroke	815	52	6.4	262	32.1	501	61.5	[33]
	Controls	430	17	4	146	34	267	62	
Chile	Hypercholesterolemia	116	25	21.6	78	67.2	13	11.2	[34]
	Controls	NA	NA		NA		NA		
China	Blood pressure	608	297	48.8	237	39	74	12.2	[35]
	Controls	616	348	56.5	216	35.1	52	8.4	
Present study	CAD	200	54	27	124	62	22	11	
	Controls	200	30	15	152	76	18	9	
**Country**	**Disease**	***n***	**Homozygous Wild Type**	**%**	**Heterozygous**	**%**	**Homozygous Mutant**	**%**	**Reference**
rs1529729									
Iran	CAD	170	43	25.44	103	60.36	24	14.2	[36]
	Controls	104	29	28.43	44	42.16	31	29.41	
Sweden	Cardiovascular	5084	1610	31.7	2481	48.8	993	19.5	[28]
	Controls	NA	NA		NA		NA		
Present	CAD cases	200	18	9	154	77	28	14	
study	Controls	200	42	21	152	76	6	3	
